# IgA nephropathy: a perspective for 2021

**DOI:** 10.1007/s00281-021-00890-9

**Published:** 2021-10-27

**Authors:** Jürgen Floege, Jonathan Barratt

**Affiliations:** 1 Nephrology and Clinical Immunology, University Hospital, RWTH Aachen, Aachen, Germany; 2 Department of Cardiovascular Sciences, University of Leicester, Leicester, UK

Immunoglobulin A nephropathy (IgAN) is the commonest primary glomerular disease worldwide and responsible for a significant proportion of the global end-stage kidney disease (ESKD) burden. While IgAN was described over 50 years ago, and despite steady progress in our understanding of this clinically heterogeneous disease, there remain significant gaps in our appreciation of the fundamental disease pathways operating in IgAN. This is reflected by the paucity of disease-specific treatments currently available for patients with IgAN. Encouragingly, this is changing with more clinical trials of novel therapies being conducted in 2021 than ever before, reflecting a growing awareness of the interactions between the mucosal immune system, innate and adaptive immune responses and tissue remodelling and regeneration. In this edition, authors cover each of these areas and offer insights into how this new understanding may be leveraged to deliver new therapies for patients living with IgAN.

Firstly, Oliver Pabst and Claudia Seikrit provide an overview of the IgA immune system, describing the normal physiological role of IgA with a focus on the mucosal associated lymphoid tissues [1]. The challenges and successes of studying IgAN and evaluating novel therapies using animal models are then discussed by Renato Monteiro and Yusuke Suzuki [2]. An increasingly important focus of research in IgAN is the mucosal immune system–kidney axis, and this is addressed firstly by Kei Haniuda, Jennifer Gommerman and Heather Reich who summarises the available evidence for a contribution of the mucosal microbiome in IgAN [3] and Loreto Gesualdo, Vicenzo Di Leo and Rosanna Coppo who provides an overview of the evidence supporting mucosal immune system dysregulation in IgAN [4]. Fundamental to the generation of kidney injury is the mesangial accumulation of IgA immune complexes and Jan Novak and Hitoshi Suzuki review the contribution of changes in the pattern of IgA1 *O*-glycosylation to immune complex formation and triggering of glomerular inflammation [5]. Following IgA and/or IgA-containing immune complex deposition in the glomerular mesangium, it is important to understand the local responses that generate tissue injury as these may direct novel therapeutic approaches. The role of complement activation is covered by Nicholas Medjeral-Thomas, H. Terence Cook and Matthew C. Pickering [6], and the downstream molecular mechanisms for inflammation and scarring reviewed by Paolo Schena [7].

Two key unanswered questions in IgAN are whether IgAN is a single disease and whether it is the same disease in different parts of the world. Hong Zhang and Jonathan Barratt discuss the available literature, comparing observations across populations, and in particular how reported differences in disease behaviour might necessitate differential therapeutic approaches in different patient populations [8].

Treatment of IgAN has been largely empiric in the past, focusing predominantly on the so-called supportive therapy, i.e. generic measures to retard progression of glomerular disease, and non-specific immunosuppression. In the contribution by Thomas Rauen, Sydney Tang and Jürgen Floege, they critically discuss this in the light of recent trial data and in particular the KDIGO treatment guidelines that will be published in October 2021 [9].

Another poorly understood aspect of IgAN is its relationship with IgA vasculitis (IgAV; previously termed Henoch-Schönlein purpura), a disease largely confined to children but occasionally also observed in adults. IgAV can be clinically heterogeneous and may be manifest as a multisystem vasculitis or alternatively be completely limited to the skin, and when involving the kidneys is histopathologically indistinguishable from IgAN. In their review, Evangeline Pillebout and Cord Sunderkötter cover the current knowledge on IgAV and its therapy [10].

We conclude this edition with a final review by Peter Boor and Julio Saez-Rodriguez, who review the future of evaluation of the kidney biopsy, in particular the use of artificial intelligence, and of systems biology approaches to gain further develop histopathologic insights and a deeper understanding of the pathogenesis of IgAN [11].

Taken together, we hope to provide a comprehensive overview of IgAN starting with basic immunological aspects and ending with clinical therapies and future developments. We sincerely hope our readers will enjoy this compilation of reviews.
